# *Salmonella* Osteomyelitis of the Rib

**DOI:** 10.1016/j.atssr.2025.06.014

**Published:** 2025-07-18

**Authors:** Ka Lam Jay Hung, Hong Yee Teddy Wong, Shuishen Zhang, Shun Chan, Kin Hoi Thung

**Affiliations:** 1 Division of Cardiothoracic Surgery, Department of Surgery, Tuen Mun Hospital, Hong Kong; 2 Department of Thoracic Surgery, The First Affiliated Hospital of Sun Yat-sen University, Guangzhou, Guangdong, China

## Abstract

A 32-year-old man was treated in our unit (Tuen Mun Hospital, Hong Kong, China), for a chest wall tumor. He had initially presented to another center because of right-sided chest pain. Computed tomography detected a chest wall mass with invasion to his ribs. He was given a diagnosis of immunoglobulin G4–related disease and was started on immunosuppression. The mass regressed, but his symptoms progressed. Open rib biopsy was performed at our center. Culture grew *Salmonella* group D, and a diagnosis of chronic osteomyelitis of the rib was made. He completed a course of antibiotics and currently follows up at our outpatient clinic and is symptom free.

Acute osteomyelitis is known to be acquired by the hematogenous route, and the most common pathogen is *Staphylococcus aureus*. Chronic osteomyelitis is often caused by gram-negative bacteria.^1^ Conversely, *Salmonella* infection manifests with sepsis, typhoid fever, gastroenteritis, localized organ disease, distant metastatic infection, and an asymptomatic chronic carrier state.^2^

*Salmonella* osteomyelitis is a rare disease entity, accounting for only 0.8% of all *Salmonella* infections and 0.45% of all osteomyelitis cases.^1^ Notably, it is associated with hemoglobinopathies such as sickle cell disease and thalassaemia.^3^ An immunocompromised status would also predispose patients to *Salmonella* osteomyelitis.^3^ Usual sites of *Salmonella* osteomyelitis include the long bones, in particular diaphysis of the femur and humerus. Other sites include the lumbar vertebrae, tibia, radius, and ulna.^2^ Osteomyelitis of the ribs, first described in 1994, is exceedingly rare, with fewer than 10 cases reported.^4^^,^^5^ Here we describe a case of *Salmonella* osteomyelitis of the rib in a young man with a protracted clinical history.

A 32-year-old man had previously been in good health, with no history of thoracic surgery or chest trauma. He had well-controlled gout and depression. He was homosexual but had been sexually inactive for years. He denied illicit drug use. He worked as a construction site worker and lived with his parents.

He first presented to the medical service at another tertiary center in August 2023 because of right-sided chest pain. Index computed tomography (CT) of the thorax showed a right chest wall tumor measuring 45 mm × 92 mm × 90 mm, with destruction of his right 7th to 10th ribs. On magnetic resonance imaging of the thorax, the mass was T2 hyperintense and T1 hypointense. Differential diagnoses included tissue inflammation, tumor, and tuberculosis. The result of a screening test for *Mycobacterium tuberculosis* was negative.

Image-guided biopsy was performed in February 2024, with histopathologic examination showing 50 immunoglobulin G4 (IgG4)–positive plasma cells per high-power field, an IgG4/immunoglobulin G (IgG) ratio of 25%, and no light chain restriction. Serum IgG was within the normal range at 15 g/L (normal, 7-16 g/L). Serum IgG4 was elevated at 2.24 g/L (normal, 0.03-2.01 g/L). The serum erythrocyte sedimentation rate was elevated at 20 mm/h (normal, 0-15 mm/h). A diagnosis of IgG4-related disease was hence made in March 2024. He was started on prednisolone and mycophenolate mofetil.

His pain improved, but his symptoms recurred in July 2024. CT of the thorax showed regression in the size of his right chest wall tumor to 30 mm × 66 mm × 70 mm. Positron emission tomography–CT subsequently showed hypermetabolism and no evidence of locoregional lymphadenopathy or distant metastasis. Tumor markers were within the normal range. A test result was negative for HIV infection. Open biopsy performed in August 2024 showed no histologic evidence of malignancy. A repeated serum IgG4 measurement at that time normalized at 1.51 g/L (normal, 0.03-2.01 g/L). Immunosuppressant agents were withheld in October 2024. On recall, he experienced an episode of gastroenteritis in June 2024. His travel, cluster, and contact history was otherwise unremarkable.

He presented to our center for a second opinion because of the persistence of his symptoms. Imaging of his thorax was repeated in November 2024 (Figure 1), and it showed an aggressive chest wall lesion measuring 40 mm × 80 mm × 90 mm with erosion to the right seventh to ninth ribs, the diaphragm, and indenting into the liver. An ultrasound-guided biopsy performed in December 2024 showed no evidence of malignancy or IgG4-related disease.

**Figure 1:**
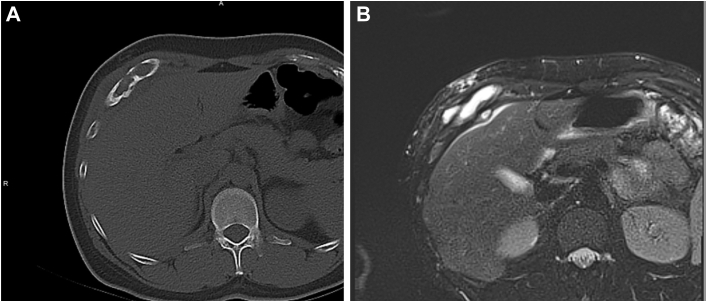
(A) Computed tomography of the thorax bone window. (B) T2-weighted magnetic resonance imaging of the chest wall.

Surgery was offered with diagnostic intent. Right partial ostectomy and open biopsy of his rib were performed in January 2024. During the operation, sequestrum and involucrum were noted over his right seventh rib. Tissue necrosis was present within the marrow cavity. Cryosection showed fibroinflammatory tissue and granulation tissue. Thorough debridement was performed. The patient’s postoperative course was uneventful. Histopathologic examination showed acute on chronic osteomyelitis with no evidence of IgG4 disease, tuberculosis, or malignancy (Figure 2). Bacterial culture grew group D *Salmonella* sensitive to ceftriaxone and azithromycin. He completed a course of antibiotics and was discharged to our outpatient clinic for regular follow-up. He was last seen in March 2025. The wound healed well, with no residual pain.

**Figure 2:**
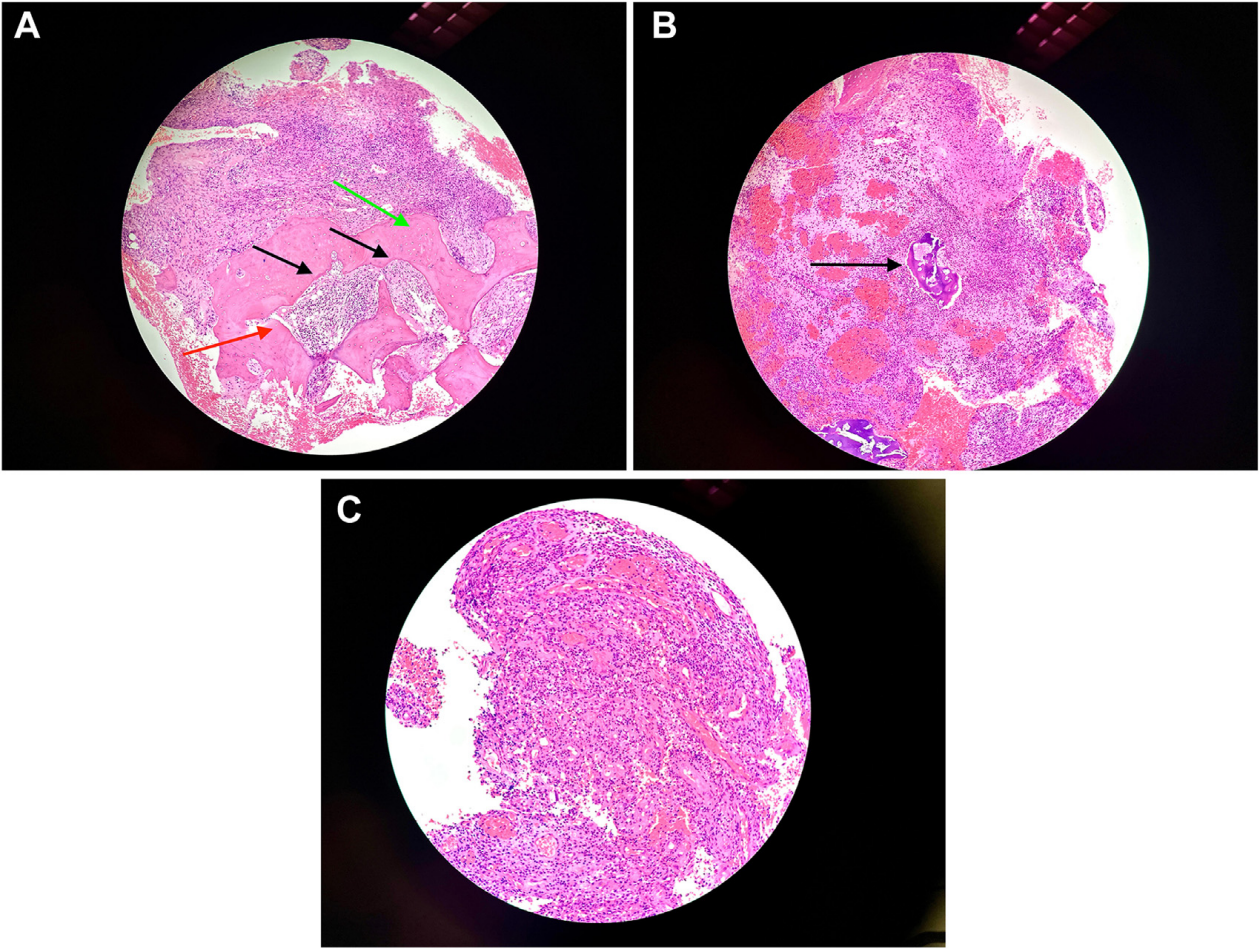
(A) Bone marrow fibroplasia and obliteration (black arrows), trabecular bone with irregular cement lines (red arrow), and lamellar bone with irregular outlines (green arrow). (Hematoxylin and eosin; original magnification ×100.) (B) Cartilage necrosis (black arrow). (Hematoxylin and eosin; original magnification ×100.) (C) A diffuse mixed inflammatory infiltrate with lymphocytes, plasma cells, foamy histiocytes, and neutrophils. (Hematoxylin and eosin; original magnification ×200.)

## Comment

*Salmonella* is an unusual organism to cause osteomyelitis, and *Salmonella* osteomyelitis of the rib is exceedingly rare.^1^ There are only a handful of case reports available of this disease entity.^5^ Although diabetes is the most common risk factor, in some instances no risk factors have been identified.^5^ Our patient’s condition was initially managed as IgG4-related disease, but his symptoms persisted. Only open surgical biopsy aided in the final diagnosis despite advances in diagnostic imaging since 1994, when the disease was first described.^4^ No case series studies have been performed on the timing of open biopsy, so one must exercise a high degree of vigilance and review the treatment direction when a patient’s symptoms persist.

Here we postulate that this patient had *Salmonella* gastroenteritis in June 2024. Transient bacteremia and a hematogenous deposit at a focally destructed nidus at his ribs coupled with immunosuppression led to his osteomyelitis. Alternatively, he could have had sporadic *Salmonella* osteomyelitis, and the diagnosis of IgG4-related disease was wrongly made with inadequate sampling and overestimation of the IgG4/IgG ratio during the index biopsy. Symptoms of rib osteomyelitis were later masqueraded by immunosuppression.

In summary, this was a young man with a chest wall mass that was initially managed as an IgG-4 related disease. His symptoms progressed despite immunosuppression, and only a repeat open biopsy revealed that he had *Salmonella* osteomyelitis of the rib. Our case highlights the importance of maintaining high clinical vigilance in patient who has a protracted history and is immunocompromised.
